# Lived experiences of refugee women with vaginal fistula in Nakivale and Oruchinga refugee settlements, Isingiro District, Uganda

**DOI:** 10.1186/s12905-024-02926-2

**Published:** 2024-02-02

**Authors:** George Opong, Everd Bikaitwoha Maniple, Caroline Noel Agabiirwe

**Affiliations:** 1 Faculty of Health Sciences, Uganda Martyrs University, Kampala, Uganda; 2 School of Medicine, Kabale University, Kabale, Uganda; 3 Independent researcher, c/o Uganda Martyrs University, Kampala, Uganda

**Keywords:** Lived experiences, Vaginal fistula, Refugee women, Psychosocial, Coping, Phenomenology

## Abstract

**Background:**

Vaginal fistula (VF) affects 2–3 million women globally, with the majority in Africa. In Uganda, it’s 2%, with western Uganda having the highest prevalence. Major predisposing factors for refugee women include health system breakdowns and sexual violence during conflict. VF has severe consequences for women, relatives, and communities. There’s limited information on lived experiences among refugee women with VF, and there’s a need for quality prevention, treatment, and social reintegration strategies. This study aimed to understand the physical, psychosocial, and economic impacts of VF on refugee women in Nakivale and Oruchinga settlements and their coping mechanisms.

**Methods:**

Ten refugee women with VF were interviewed using qualitative study design, utilizing Social-Ecological and Transactional Models for data collection, analysis, and discussion.

**Results:**

Ten refugee women aged 24–50 years with or who had experienced VF participated in the study. They lived with VF for at least 2–15 years and had multiple stillbirths. Obstetric Fistula (OF) was the leading cause, followed by rape and cancer. Post-fistula, they faced social discrimination, emotional disturbances, survival difficulties, poverty, and lack of support. They struggled with stigma, social isolation, and marital sexual challenges.

**Conclusion:**

Refugee women experience physical, emotional, financial, social, and sexual trauma due to VF. Discrimination and stigmatization from loved ones and society lead to isolation, depression, and suicidal thoughts. Despite successful repair, their social and emotional healing remains a burden for their lives. There is a need to provide a supportive environment for VF survivors.

**Supplementary Information:**

The online version contains supplementary material available at 10.1186/s12905-024-02926-2.

## Background

Vaginal fistula (VF) is a common condition among women globally, characterized by an abnormal opening between the vagina and another nearby organ, such as the bladder, colon, or rectum, allowing uncontrolled leakage of urine and/or stool through the vagina [1]. It has devastating effects on women’s physical and psychological health, and has significant negative socio-economic consequences on the affected women and their families. It can lead to neurological disorders, orthopedic injuries, bladder infections, painful sores, kidney failure, and infertility [2]. Many VF survivors go unreported and live marginalized lives in poverty and die in humiliation and dishonor [3]. It mainly affects young, poor, and uneducated women in remote communities with poor maternal health care. VF survivors face neglect from family and the community, are often childless and seen as outcasts [4]. VF frequently results from injuries obtained during childbirth, violent sexual assault especially during armed conflict, surgery, infection, cancer, or radiation treatment [5]. It is a life-long disability that affects a woman’s productivity at household and community levels [2, 6–8]. Most (> 90%) VF survivors have also lost their baby during birth [9] and are abandoned by their spouses in most African countries [10]. Broken health systems e.g. during crises, leading to obstructed access to health care, lack of skilled personnel, and inadequate infrastructure and supplies, increase the risk of VF [11–17].

VF prevention and management tends to focus on the physical problems, neglecting issues of social support and community re-integration. VF survivors often struggle to restore their “marital value”, loss of which led to isolation and loss of identity. They use problem-focused coping strategies such as homemade absorbent pads to manage incontinence, frequent bathing and use of herbs and smoke to mask odour [18–20]. They also use surgical treatment, remarriage, and transferring to new communities to gain acceptance [21]. They have been referred to as ‘modern lepers’, using faith to cope [22].

VF affects an estimated 2–3 million women, primarily in Sub-Saharan Africa and Asia [23]. In Uganda, it affects 2% of women aged 15–49 years, with western Uganda having the highest prevalence of 4% [1], although the trend shows a decline in prevalence due to improved training and facilities [24]. VF surgical treatment is part of the package of free health services offered in Uganda.

However, VF prevalence, consequences and lived experiences among refugee women have not been documented widely globally or in Uganda. Uganda hosts the largest number of refugees in Africa, most of who have fled armed conflict in the neighbouring countries. Globally, there are over 32 million refugees and asylum seekers [25–27]. Refugees face significant physiological, social, and psychological problems due to their situation. They often stay in isolated areas during transit and resettlement, leading to low self-esteem, depression, and their consequences. They struggle to be accepted, to engage in social and economic activities, and experience physical injury, feelings of powerlessness, emotional breakdown, despair, divorce, social capital erosion, and lost years of health [18, 28]. The present study aimed to explore the socioeconomic, physical, psychological lived experiences and coping strategies of refugee women with VF in two refugee settlements in south-western Uganda, namely, Nakivale (pop. 136,399 on 71.3 mile^2^) and Oruchinga (pop. 8176 on 8.4 mile^2^). It aimed to identify the lived experiences of refugee women with VF who have been diagnosed but not yet undergone VF repair, those whose repair has been successful, and those whose repair attempts have, so far, been unsuccessful, and their coping mechanisms. The findings may benefit individual refugee women, their families, communities, and humanitarian society in making informed decisions on extra services for VF survivors. The study also contributes to knowledge by exposing the additional challenges faced by refugee women with non-obstetric VF. The study aims to provide informed recommendations for intervention and screening measures at transit and entry points to ensure early care for VF survivors.

### Theoretical framework

The socio-ecological model and the transactional model of stress and coping are two theories that help explain the experience of VF from different perspectives [28, 29].

The socio-ecological model, revised in 2005, focuses on the interaction between personal, situational, sociocultural, and environmental factors, including the built environment. It helps to understand the influence of intrapersonal and interpersonal behavioural factors on women’s health and social circumstances, as well as socio-cultural and environmental factors such as policies on the occurrence and consequences of fistula.

The transactional model of stress and coping is used to understand the risk factors that affect refugee women and girls in their communities and may be used to design interventions to address the reduction of risks at different levels. This model provides a more holistic approach to health interventions that not only targets individual health needs but also addresses the need for social change. The transactional model of stress and coping shows that VF stressor demand is made by the internal or external environment that upsets the survivor’s balance, affecting their physical and psychological well-being and requiring action to restore balance. Refugee women perceive VF stressors as person environment transactions, which depend on the impact of the external stressor. They evaluate not only the features of the stressful situation but also what they can do about it. They assess their perceived ability to change the situation and manage their emotional reaction to the threat through actual coping strategies.

The main coping efforts in this model are problem-focused and emotion-focused coping strategies. Problem management strategies, which are more adaptive for changeable stressors, include active coping, planning problem-solving, information seeking, and use of social support. Emotion-focused coping efforts, which are more suitable when the stressor is unchangeable, are directed at changing the way one thinks or feels about a stressful situation [30]. Overall, the socio-ecological model and transactional model of stress and coping offer valuable insights into the experiences of refugees with VF. By incorporating these models into public health practice interventions, researchers can better understand and address the complex interplay of biological, psychological, social, cultural, economic, and political factors affecting the experiences of these individual.

## Methods

### Study area

The study was conducted in two long-standing refugee settlements. Nakivale refugee settlement was established in 1958 and Oruchinga in 1961 [31]. The two settlements are located in present-day Isingiro District. Women and girls comprise almost half (49.6%) of Nakivale’s total population, with 52.5% being girls below 18 years [31–33]. The refugees at Nakivale and Oruchinga come from 13 nationalities, with the majority being from the neighbouring Democratic Republic of the Congo (DRC) [32]. Others are from Rwanda, Burundi, Somalia, South Sudan, Ethiopia and Eritrea.

### Study design

The study was of qualitative design and a phenomenological inquiry. It investigated the double tragedy of being a female refugee and having a VF. Phenomenological methods are useful in challenging structural or normative assumptions and adding an interpretive dimension to research [34]. Phenomenological studies have influenced policy changes in public health, reproductive health, mental illness, and adolescent health [35–41].

### Data collection methods

The study received ethical approval from Uganda Martyrs University and administrative permission from the management of each settlement. Informed written consent was sought from each participant before the interview Fifteen eligible refugee women with VF, irrespective of previous repair and outcome status, were purposively identified through medical records at the respective settlement health facilities and the women were located with the help of settlement health workers. Only ten of the fifteen identified eligible participants consented to participate in the study. Four participants had had successful VF repair, four had failed repair attempt, and two who had never undergone repair.

An in-depth interview was held with each participant and data collection stopped at the saturation point, when no more new information was being generated. The interviews took place from February to March 2018 and the participants were interviewed either from their homes or (8) or health facility where they felt free to express themselves without interference (2). The homes of fistula patients were located by fistula focal persons in each village working under Medical Teams International (MTI), an international health implementing partner for UNHCR. Eligible participants were refugee women with VF, aged 18 years or older, living in Nakivale or Oruchinga refugee settlements, who had VF before coming to Uganda. Women with VF but who were not registered as refugees in the two settlements were excluded. Interview guides were administered by female research assistants trained in data collection methods for traumatized women. The shared experiences were audiotaped and manual notes were taken simultaneously. A female refugee interpreter was used for participants who could not express themselves in English. The audio recordings were transcribed and transcripts later compared with the notes. Confidentiality was ensured through use of pseudonym initials to protect participant identities.

### Data analysis

The study employed six of Colaizzi’s 7-step method to analyse the data [42]. The final step, member-checking, was not done in order to minimise the risk of exposing the participants’ identities. Interpreters from the nationalities of each participant helped to ensure completeness and accuracy of translation of the transcripts.

## Results

The ten refugee women with VF interviewed were aged 24–50 years, having had VF for periods ranging from 2 to 15 years. Three were Somali, three from DR Congo, two from Burundi and two from Rwanda. All had been married before, with five divorced and two separated from their spouses during flight. Most had no children due to stillbirths or one child, but three had a child. Of the 10 VF survivors, 5 attributed Obstetric Fistula (OF) as the underlying cause, 4 reported being victims of armed sexual assault and 1 case was linked to cancer (see Table 1).

**Table 1:** Socio-demographic characteristics of the participants

Pseudonyms initial^**a**^	Age	Nationality	Duration with VF	Cause of VF	Marital status	No. of children	State of VF
N	32	Congolese	Since 2013	Gang Rape	Married	3	Failed repair
A	24	Somali	Since 2010	Obstetric	Divorced	0 (Still birth)	Failed repair
M	39	Rwandese	Since 1995	Obstetric	Divorced	0 (Still birth)	Failed repair
N	50	Congolese	Since 1990	Obstetric	Married	0	Still with the fistula
N	43	Burundian	Since 2011	Rape	Married	6	Successful repair
M	45	Rwandese	Since 1996	Obstetric	Divorced	3	Successfully repaired
A	41	Somali	Since 2015	Rape	Widow	1	Successfully repaired
B	50	Congolese	15 years	Obstetric	Married	3	Not yet repaired
S	42	Somali	Since 2010	Rape	Separated during war	7	Successfully repaired
K	24	Burundian	Since 2015	Cancer	Divorced	1	Irreparable

^a^The pseudonym initials used do not represent the real names of the participants

The study identified 20 significant themes from transcripts, which were then grouped into five major themes: Poor maternal health care services (Poor infrastructure and Poor/no health facilities), Sexual abuse (Rape and Vaginal piercing Rape) Social discrimination (Deserted, Neglected and Divorced), Emotional disturbances (Shame, Shunned, Depressed, Stigma, Suicide and Pity), and Poverty and dealing with a difficult situation (Un-employment and increased expenses) as shown in Table 2.

**Table 2:** Shows the common initial codes, Higher level codes axial themes and the essential themes

Primary codes (tally)	Secondary codes	Axial codes/Sub themes	Themes
**How you got the problem /Causes:**			
Difficult delivery (I) Prolonged labour (III) Removed dead baby (I)	Obstructed labour Stillbirth	Poor/no health facilities	Poor or no maternal health care services
After the operation (II) Instrument delivery (I)	Instrumental		
Cancer (I)	No screening services		
No transport (II) Far Health centre (II) No roads (Fear to travel to Hospital (II)	No or poor roads Far or no Hospitals, no staff to attend to them	Poor infrastructure	
Rape (III) Pierce private part using instruments (II)	Forced sex Vaginal piercing	Rape	Sexual abuse
**Onset**			
Seeing blood passing through my anus and vagina (I) Seeing stool pass through my vagina (I)	Unusual experience	New experience	New experience
Sudden unexpected onset of blood (I) Sudden onset of urine (II)	Unexpected		
I cannot even tell what caused this problem I was later old	Unknown problem		
**Effect**			
Living with family members and community			
Husband abandoned me: (I) Took my children away (I) Hide/Isolate self from others (III) No longer considered a family member (I)	Family neglect Divorced	Neglected/Loss company Divorced	Social discrimination
People abandoned me: (IIII) Calling me bad names: (I) People avoid me due to smell: (III) Avoid people: (II) People complain of bad smell: (I	Neglected		
Waste of family money: (I) Arrested because of mistaken identity: (I)	Increased expenses		
No value in me: (I) Problems double: (I) Life becomes useless, not worth living (II) Hell on earth: (I) Physical shock: (I) Mental shock: (I) Shame: (I) Sadness and guilt: (I)	Loss of self-worth Hiding suffering Felt labelled Labelled criminal Guilt Depression	Suicide Depressed Shame,	Emotional disturbances
Interference with sex: (III) Mess in bed and clothing’s: (Terrible smell: (III) Bad smell: (IIII) The woman who defecate on herself: (I)	Feeling Dirty Dirty	Deserted, Shunned, stigma	
Experience during movement: Difficult movement: (III) Chest away by people because of bad smell: (III) Abuse of being smelly: (II) Perceiver: (I)	Abandoned Insult	Neglected and Depression	
Coping with economic and spiritual life			
Difficult to do business: (Difficulty in working or maintaining: (III) Fear of crowd: (I) Stopped all social life: (I) Collapsing business: (I) Customers chest away by smell: (I) Difficulty in getting a job: (I)	Difficulties in selling Difficulties in getting and maintaining a job	Un-employment	Poverty and Dealing with difficulties for surviving
Change in eating habit: (I) Increased demand for water and soap: (II) Increase economic burden: (I)	Increased expenditure	Increased expenses	
Life as a refugee:			
Food ration not adequate: (I) Miss hot meals served at the reception centre: (III) Difficult to attain a ration card long bureaucracy: (IIII) Difficult to stand online: (III) Miss monthly food ration: (II)	Lengthy ration collection waiting time, Long refugee registration process, Long food distribution duration Inadequate food ration	Long Bureaucracy	
Expected life Donation of pads: (III) Help to get ration card: (I) Help to get ration food: (II)	Hygiene need Support	Survival	
Coping			
Privacy to clean-up myself: (II) Not minding the smell: (III) Drinking less water: (II)	Hiding Adopting to smell	Coping	Pity Coping
Going back to parents: (II) Sympathy from other (IV) Pity from people: (III) Sympathy: (I) Fear of praying with people: (I)	Sympathy from the community	Pity	
Putting all my trust in God: (II) Crying telling God: (I) Increase church-going: (I)	Sympathy and trust Religious	Empathy from Community Feeling more religious	

### Lived experiences with VF

The participants reported that VF brought them shame and stigma in their society. They experienced social discrimination. They used words like feeling “shunned” “persistently avoided”, “shame”, “deserted”, “neglected” and “divorced”. They feel they have been “isolated” from and “abandoned” by their family members and society. The survivors reported that they often feel “denied” by those who have shown them love and care, losing their respect and value in society. They feel “fearful” of attending social gatherings and encountering people. They feel “worthless” due to VF consequences, leading to a shameful life in the community and in bed, due to failure to perform normal recreation, and have a feeling of being “cursed” among women (see Additional file 1).

‘*I was married for 18 years but immediately I got this problem he (Husband) abandoned me for another woman because he could not tolerate the smell and the wet beds every time. He said he has spent a lot of money on me in hospital bills and he still needs a baby, and there is no more value in staying with me as his wife’.* (M, Rwandan refugee).

Similar consequences were experienced by tthree other participants:

‘*After the problem, my husband decided to marry another wife since he could not withstand the smell and the wet beds. He even took away my children from me’* (M, Burundian refugee).

*‘After getting this problem he couldn’t tolerate it. He chased me away from his home, that I should go back to our home until I get healed and regain normal life as before’* (A, Somali refugee).

*‘I was married for nearly 5 years but when I got this problem my husband abandoned me for another woman*’ (N, Burundian refugee).

Feelings of shame were a prominent report among the women.

*‘It is such a disturbance and a shameful moment to my normal life, …. This forced me to go back home to my parents since I could not tolerate the shame and fear of failing to perform my duties and function as a woman’. (*K, unrepaired Congolese refugee*).*

‘*I had a terrible smell as I didn’t have any time to bathe and there was no soap to use or enough water, no additional clothes to keep changing into. I could see people covering their nose whenever they were with me. … one passenger got out of the bus because she couldn’t travel in a smelly taxi’. (*N, successfully repaired Sudanese refugee*).*

Apart from shame, feelings of loss of self-worth and guilt were reported. ‘*… they started abusing me, calling me all sorts of names, as a woman who has been cursed .., I felt sinful and not human enough, not worth living…” (*S, gang raped Somali refugee, still unrepaired).

‘*Though my husband decided to marry another woman, I felt it was o.k., … I felt guilty because of my husband being wet every night from my urine, I feel small and worthless’ (*M, Burundian refugee, still unrepaired).

The participants’ narratives were replete with multiple accounts of personal emotional disturbance:

‘*I was last happy in my life when I was still a girl’. (*M, Rwandan refugee*).*

Four of the survivors experienced suicidal thoughts at least once:

‘*Life became useless, even, my normal life became less enjoyable (*tears rolling her eyes*) even my husband does not even want me, is life now worth living?’ (*K, Congolese refugee*).*

*‘I had already taken a rope to kill myself, but my thoughts told me to come to the nearest border’. (*N, gang-raped Congolese refugee*).*

The personal predicaments of the women were aggravated by their status as refugees. Right from the border points of entry into Uganda, through the transit centres to the settlement sites, the women felt a lot of social pressure due to their VF condition:

*‘… policemen would chase us away because they could not bear the smell of faeces and urine. Even the fellow refugees … chased us out because of the unbearable smell. …we were put in a big tent …. and I was chased away … to sleep outside, where I was badly bitten by mosquitoes. (*N, Sudanese refugee*).*

Moreover, the VF survivors experienced discrimination on the basis of their history. For example, rejection was enhanced if one had been raped, because this was also associated with having acquired HIV. The in-laws wanted her out of the family since they believed that she was going to spread the disease to their brother.

*‘The worst words of abuse came from my in-laws. … They forced my husband to abandon me, saying that I was already infected with HIV by the rebels’ (*N, gang-raped Congolese refugee*).*

Negative feelings were worsened by a sense of hopelessness because help was not immediately forthcoming. The organisations in which the VF survivors placed their hopes did not show much concern over the problem.

*‘Even the fellow refugees with whom we were placed in the same room would chase me out because of the unbearable smell’. (*K, Sudanese refugee with VF due to cervical cancer*).*

Hopelessness was enhanced by the awareness that the likelihood of successful repair dwindled with every failed attempt.

*‘I have been to many hospitals here in Uganda and also hospitals back home but they just had bad news for me. The Doctors tell me that the problem I have cannot be repaired anymore, I have no hope in life. ... People are tired of me., Life is not worth living. All that is on my mind is, I think if I die that would be fair to end this suffering’.* (A, Somali refugee with repeated failed repair attempts).

Obtaining ration cards and monthly food rations was also a challenge, due to long queues, leading to missing food rations.

*‘It was really difficult to attain a ration card and supplies. The long queues, waiting time and the back-and-forth referral day after day… I failed to attain my ration card’. (*M, Rwandan refugee*).*

‘*It was hard to get the food ration card and even making lines and stand the whole day while leaking like that.’ (*N, Burundian refugee*).*

### Coping mechanisms

As the participants faced difficulties, they struggled to maintain their physical health and social lives through various mechanisms. Some looked for jobs. Others sought assistance from sympathetic family members or religious organisations and faith-mates. Some of the women with VF sent the neighbours’ children and well-wishers to help them collect the food rations, although they were sometimes rejected by the food distributors(see also Additional file 1).

*‘I always send my neighbour and well-wishers but this is not always a success. Sometimes, they are refused my food ration’. (*A, Somali refugee*).*

Some attempted business while others resorted to begging and being dependent on family members and well-wishers.

‘*I sold second-hand clothes. However, I could not stand for long because I had to be on and off, to change my clothes. One time, they stole 20 pieces of clothes from me while I had rushed to change diapers. …, I had to quit the business. I tried to work as a housemaid …but the family chased me after just a week, abusing me for being unhygienic and smelly’. (*A, Somali refugee*).*

## Discussion

The study aimed to provide insights into the lived experiences of refugee women with VF and the coping strategies that they adopted while in exile. It highlights the fact that living with VF is a complex and multifaceted and multi-level experience at the individual, family, community and entire societal levels. The findings show that the VF survivors faced social discrimination, isolation, broken relationships, and emotional disturbance like loss of self-worth and suicidal ideation. They experienced poverty as a consequence of the illness.

### Social discrimination and emotional disturbances

Social discrimination and loss of dignity have been observed in previous similar studies [10, 36, 43] where women with VF were considered social outcasts and labelled as “shameful”. The study reveals that individuals with VF face significant discrimination from the community and auto-stigma. This is consistent with many studies on fistula in African women [41, 44]. The participants’ perceived themselves as being “cut off” from the community, similar to what was found in a previous study [45]. Other studies have shown that VF is often associated with evil, sinfulness, or punishment from an angry God [8, 46]. The social reactions towards people with VF are influenced by fear of having a bad smell and being included in the punishment by God. Since most of the participants with VF also had no children, VF is associated with childlessness. The relationship between these facts and their community interpretations are quite complex. These are compounded by the refugee status which might also have its additional interpretations in the community setting. VF survivors are the bogeymen and scapegoats of their communities.

Studies have shown that women with VF experience social rejection to the extent that they are forced to live as outcasts, not allowed to handle food, cook, or even pray [8, 47, 48] However, these had not been studied among refugee women. Some studies had already found that urban refugees, particularly Congolese and Somali adolescents, reported cases of discrimination from health care workers and service providers of other refugee services [49]. However, there was no similar report about refugees in rural areas like the ones in the present study. Despite policies and advocacy for reducing VF, most focus is on surgical management to reduce leakage and incontinence. However, socio-economic inclusivity has not been widely addressed. As a result, women with fistula still face stigma and discrimination.

The study participants experienced emotional disturbances due to community rejection They experienced rejection from loved ones and family members.This is consistent with previous studies, where 53.3% of the women reported rejection [21].

### Coping strategies

The study also revealed that the participants used two broad forms of coping strategies: (1) problem-based coping strategies and (2) emotional-based coping strategies: seeking support from their families and neighbours. Problem-based strategies aim to eliminate the source of the smell while emotion-based strategies aim to improve personal resilience in the face of isolation and shame. A study in Tanzania [43] found that successful surgical repair improved VF survivors’ chances of community acceptance and reintegration. This was observed in a similar study [50] that showed that 75% went back to their previous occupations following successful surgery. Therefore, post-surgery care of VF survivors should focus on holistic recovery involving physical and emotional healing as well as occupational rehabilitation for complete social reintegration. Communities and families with VF survivors also need support in order to reabsorb them after recovery. There is need for public health education to prevent other women from developing VF and for the community members to appreciate that VF is both preventable and treatable, and should not lead to discrimination. There is need for strengthening health services so that mothers with high risk of obstructed labour can be identified early and given additional attention, and that complications can be detected and managed early. Stronger measures to prevent the use of sex as a weapon of war should also be put in place to prevent belligerents from sexually exploiting the civilian population and causing problems like VF.

### Suggested areas for further research

Future research should focus on community integration after VF repair, exploring men’s experiences of divorce due to VF, conducting a community-based study to understand community perspectives on female genital fistula, and examining the impact of obstetric fistula on survivors’ families. Four of the ten participants reported rape as the causes of their vaginal fistula. In a non–war zone the cause of fistula is rarely related to sexual abuse, but it more frequently it can be determined by post-coital laceration. Therefore, future research should investigate more specifically whether even in the African population the extremity of age and the execution of specific sexual positions are risk factors of vaginal trauma as recently reported in the literature [51].

### Strengths and limitations of the study

#### Strengths

Qualitative phenomenological research aims to describe the “lived experience” of a phenomenon, providing a rich description of it by those who have experienced it. This study provided an in-depth understanding of individual experiences of the phenomenon, providing rich data from the experiences of individuals. This study provides an in-depth understanding of individual phenomena and provides rich data from the experiences of individuals.

#### Limitations

The study on VF among refugee women in rural settlements of Nakivale and Oruchinga has several limitations, including recall bias, a purposive sample, and the lack of proof for the phenomenological studies [52, 53], generalizability, and objectivity of the findings. Although the credibility of the study was identified, generalisation is cautioned because of the small number of participants [54]. The participants were mostly from refugees who fled their countries due to civil war, which may not be representative of refugees who fled due to other causes like natural calamities.. Despite these limitations, the study provides valuable insights into the experiences of women with VF and serves as a foundation for future research on this topic.

## Conclusion

This study explores the lived experience of refugee women living with VF. The study’s findings are consistent with other studies on VF, but none had been conducted in a refugee setting. It highlights the significant impact of VF on women’s physical and psychological health and quality of life. There is need for community members, humanitarian aid workers and health workers to improve their understanding of VF and its consequences, in order to provide a supportive environment for VF survivors.

### Supplementary Information

**Additional file 1.** Participant testimonies.

## Data Availability

Data can be made available on request from the corresponding author.

## Abbreviations

MTI: Medical Teams International
OF: Obstetric Fistula
OPM: Office of the Prime Minister
UNHCR: United Nation High Commission for Refugees
VF: Vaginal Fistula
