# Supplementary material for: Lived experiences of refugee women with vaginal fistula in Nakivale and Oruchinga refugee settlements, Isingiro District, Uganda
Source: BMC Womens Health. 2024 Feb 2;24:85. doi: 10.1186/s12905-024-02926-2 (PMC10836010; doi:10.1186/s12905-024-02926-2)
Supplement: Supplementary file 1 — Additional file 1. Participant testimonies. [file 12905_2024_2926_MOESM1_ESM.docx]

**Additional file 1: Participant testimonies**

1. *Causes of Vaginal Fistula(VF)*

Of the 10 participants interviewed, 5 reported that they got a VF through a difficult and prolonged delivery.

1. *Prolonged and difficult labour*

*‘I got the problem after a difficult delivery of my firstborn. The baby was delayed for a very long time at home while I was in labour since I could not get transport to the nearest health center. They even removed the baby from me when it was already dead.’* (M, Rwandan refugee)

‘*I experienced the problem at the time of delivery, I spent two days in labour in one of the refugee settlements I was staying in Tanzania. The Ambulance took me to the hospital later, but I was told I could not deliver normally. So, I was to be operated on, but it was after the operation that I started experiencing a constant flow of urine.’* (M, Rwandan refugee)

*‘It happened after delivery of the first baby after spending two days in labour where they had to pull the baby out using an instrument’.* (N, Congolese refugee)

*‘When I was giving birth, the child was forced out. After that, I got that problem and doctors tried to help me but there was no change at all, and they told me that I needed an operation, and I was operated on.’* (B, Congolese refugee)

1. *Sexual abuse*

‘*I was cooking when the rebels attacked us. They used boiling water to burn some of my friends. For me, I was raped to the point that I thought I was dead. I lost all senses, and when I woke up I found urine dripping constantly down my private parts without control and with a lot of bleeding and injuries. I felt a lot of burning as urine dripped through my private parts.’ (*N, Congolese refugee)

‘*It was during the wartime, I was travelling from my town to a nearby place, and we were stopped by the militia who ordered the drivers to get all the women out and drive away. We were then taken to the bush, and the two of us were raped by seven men.’* (A, Somali refugee)

‘………...*that evening, the rebels came and tortured us severely. They cut me on the legs, and they even pierced my private parts using sharp objects. I was unconscious, but when I woke up, I got myself with severe pain in my private parts, and a lot of other wounds and urine was flowing constantly without any control.’* (S, Somali refugee)

*‘The rebels came and captured both my husband and me and they forced us to carry luggage and when we reached the forest, they dragged me to the forest and raped me’*. (N, Burundian refugee)

1. *Cancer*

*‘It all started by seeing blood passing through my anus and private parts, and later I started seeing stool passing through my private parts, it was an unexpected scene to me. I couldn’t even tell what the cause of this problem was. I was later told that the problem was a result of cancer.’* (K, Burundian refugee)

1. *Lived experiences with VF*

The participants interviewed viewed VF as a disease of shame that is highly stigmatised by society and the people around them.

*i) Social discrimination*

The participants interviewed viewed VF as a disease of shame that is highly stigmatised by society and the people around them.

‘*I was married for 18 years but immediately I got this problem he (Husband) abandoned me for another woman because he could not tolerate the smell and the wet beds every time. He said he has spent a lot of money on me in hospital bills and he still needs a baby, and there is no more value in staying with me as his wife.’* (M, Rwandan refugee)

Three other participants recounted similar confessions:

‘*After the problem, my husband decided to marry another wife since he could not withstand the smell and the wet beds. He even took away my children from me’* (M, Rwandan refugee).

*‘After getting this problem he couldn’t tolerate it. He chased me away from his home, that I should go back to our home until I get healed and regain normal life as before.’* (A, Somali refugee)

*‘I was married for nearly 5 years but when I got this problem my husband abandoned me for another woman.*’ (N. Congolese refugee).

*ii)Shame and loss of self-worth*

*‘It is such a disturbance and a shameful moment to my normal life, I started seeing faeces passing through my vagina. Every time I tried to have sex with my husband the whole bed got messed up with faeces and with a terrible smell. This forced me to go back home to my parents since I could not tolerate the shame and fear of failing to perform my given duty and function as a woman.’* (K, Burundian refugee)

‘*I had a very bad experience with this problem, but I had nothing to do. I had to persevere. I had a terrible smell as I didn’t have any time to bathe and there was no soap to use or enough water, no additional clothes to keep changing into, so I could see people covering their nose whenever they were with me. I remember there was one passenger who got out of the bus because she couldn’t travel in a smelly taxi.’* (N, Congolese refugee)

‘*I was physically and mentally shocked, in the village they started abusing me calling me all sorts of names, a woman who has been cursed because of her evil ways, and this brought a lot of guilt to myself. I felt worthless, I felt sinful and not human enough not worth living among the community member's.’* (S, Somali refugee*)*

‘…. *though my husband decided to marry another woman, I felt it was o.k., as to myself I felt guilty because of my husband being wet every night from my urine, I feel small and worthless.’ (*M, Rwandan refugee)

1. *Emotional disturbances*

The participants shared experiences of emotional disturbance due to VF, including feelings of rejection, divorce, depression, stigma, and suicide.

‘*It’s really sad to have this problem because I was last happy in my life when I was still a girl but after getting this problem, I was staying with my husband who truly married me and had much love for me. We even wedded at the church without problems but when I got this problem, he decided to leave me and would not even want to be near me saying it’s shameful to call me or even associate with me as a person he knows.’* (M, Rwandan refugee)

*‘After getting this problem, my problems doubled, as every time my clothes are dirty because urine passes through, nonstop. I fear to go near other people and I isolate myself and hide from the neighbours and friends.’* (N, Burundian refugee)

‘*I was physically and mentally shocked because, in the village, they started abusing me calling me bad names like:” look at this woman who urinates on herself like a child, I think she is mentally ill.’* (S, Somali refugee)

*‘Everyone who received us including the border policemen would chase us away because they could not bear the smell of faeces. Even the fellow refugees with whom we were placed in the same room chased us out because of the unbearable smell. …we were put in a big tent, during the first night …. everyone abused me for being smelly and I was chased away … to sleep outside, where I was badly bitten by mosquitoes.’* (K, Burundian refugee)

Four survivors recounted suicidal thoughts at least once after developing a VF,

‘*It was such a disturbance to my normal life, I started seeing faeces passing through my vagina, every time I try to have sex with my husband the whole bed gets mixed with faeces and with a terrible smell. This forced me to go back home to my parents since I could not tolerate the burden. Life became useless, even, my normal life became less enjoyable (tears rolling her eyes) even my husband does not even want me, is life now worth living?’* (K, Burundian refugee)

*‘Everyone abandoned me after knowing that I was raped. The worst words of abuse came from my in-laws. It was real hell on earth. I was no longer considered as a family member. They forced my husband to abandon me, saying that I was already infected with HIV by the rebels and that I was so smelly and not worth staying in their family, that I was a taboo to the family. The same night I fled to Uganda, I had already taken a rope to kill myself, but my thoughts told me to come to the nearest border.’* (N, Burundian refugee)

*‘It was a difficult situation during movement, everyone who received us including the border policemen would chase us away because they could not bear the smell of faeces. Even the fellow refugees with whom we were placed in the same room would chase us out because of the unbearable smell.’* (K, Burundian refugee)

*‘I have been to many hospitals here in Uganda (Kitovu Hospital, Mbarara Hospital and Mulago) and hospitals back home in Somalia but they just had bad news for me. The Doctors tell me that the problem I have cannot be repaired anymore, I have no hope in life. Living with this urine and stool is just horrible. I don’t even have money to buy diapers. All I have to do is to borrow money. People are tired of me., Life is not worth living. All that is on my mind is, I think if I die that would be fair to end this suffering.’* (A, Somali refugee*)*

*3. Coping mechanisms*

Participants noted difficulties maintaining their physical health and social lives. Some women with VF were helped by neighbours to collect food rations, although distributors sometimes rejected them.physical and social health. Some reported looking for jobs, and assistance from family members, religious organisations, and faith-mates. Some reported being helped by neighbours to collect food rations, although distributors sometimes rejected them.

*‘It was really difficult to attain a ration card, the long bureaucracy, the long waiting time and the back-and-forth referral day after day…. it was difficult for me to attain a ration card, so there is nothing I get as a refugee since I failed to attain my ration card.’* (M, Rwandan refugee)

‘*It was hard to get the food ration card and even making lines and standing the whole day while leaking like that. It was hard, though you change. Sometimes it burns you and sometimes they tell you that “Today, no work. Come tomorrow” and on coming back, they tell you to come yet another day and even on that given day, they don’t work on you.’* (N, Burundian refugee*)*

*‘I always send my neighbour and well-wishers who sometimes help me get the food, but this is not always a success. Sometimes, they are refused my food ration.’* (A, Somali refugee)

‘*I sold second-hand clothes. However, I could not stand for long because I had to be on and off, to change my clothes. One time, they stole 20 pieces of clothes from me while had rushed to change diapers. …, I had to quit the business. I tried again as a housemaid …but the family chased me after just a week, abusing me for being unhygienic and smelly.’* (A, Somali refugee)

*‘My demand for soap and water has greatly increased. Neither I nor my husband is working. I have to wash my clothes and pads every day and, every time, this requires over half a bar of washing soap every day. Sometimes, I just give up and stay smelly. If I wash with plain water, the smell does not go away.’* (N, Congolese)

*‘As you can see this is already a collapsing bar, every time people come here to drink beer, they take only a bottle of beer and you don’t see them coming back again, I overheard from neighbours that people who come to drink from this bar feel disgusted by the smell of urine in the bar, this makes them never to come back again.’* (M, Rwandan refugee)

‘*I have nothing but there is a Somali family who sympathised with me to stay with them, but the burden to the family is much. They can’t provide for my diapers. It is the Somali community who always comes to my rescue to provide for my diapers and sometimes collect some money for my food.’* (A, Somali refugee)
